# Gender bias revisited: new insights on the differential management of chest pain

**DOI:** 10.1186/1471-2296-12-45

**Published:** 2011-06-06

**Authors:** Stefan Bösner, Jörg Haasenritter, Maren Abu Hani, Heidi Keller, Andreas C Sönnichsen, Konstantinos Karatolios, Juergen R Schaefer, Erika Baum, Norbert Donner-Banzhoff

**Affiliations:** 1 Department of General Practice/Family Medicine, University of Marburg, 35032 Marburg, Germany; 2 Department of Family Medicine, Paracelsus University, 5020 Salzburg, Austria; 3 Department of Cardiology, University of Marburg, 35032 Marburg, Germany

## Abstract

**Background:**

Chest pain is a common complaint and reason for consultation in primary care. Few data exist from a primary care setting whether male patients are treated differently than female patients. We examined whether there are gender differences in general physicians' (GPs) initial assessment and subsequent management of patients with chest pain, and how these differences can be explained

**Methods:**

We conducted a prospective study with 1212 consecutive chest pain patients. The study was conducted in 74 primary care offices in Germany from October 2005 to July 2006. After a follow up period of 6 months, an independent interdisciplinary reference panel reviewed clinical data of every patient and decided about the etiology of chest pain at the time of patient recruitment (delayed type-reference standard). We adjusted gender differences of six process indicators for different models.

**Results:**

GPs tended to assume that CHD is the cause of chest pain more often in male patients and referred more men for an exercise test (women 4.1%, men 7.3%, p = 0.02) and to the hospital (women 2.9%, men 6.6%, p < 0.01). These differences remained when adjusting for age and cardiac risk factors but ceased to exist after adjusting for the typicality of chest pain.

**Conclusions:**

While observed gender differences can not be explained by differences in age, CHD prevalence, and underlying risk factors, the less typical symptom presentation in women might be an underlying factor. However this does not seem to result in suboptimal management in women but rather in overuse of services for men. We consider our conclusions rather hypothesis generating and larger studies will be necessary to prove our proposed model.

## Background

Chest pain is a common reason for consultation in primary care and incidence varies according to setting, country, and inclusion criteria [1-3]. Chest pain can be caused by a wide range of different diseases including Coronary Heart Disease (CHD) [4,5]. Gender differences in chest pain patients finally diagnosed for CHD have been described for different clinical symptoms and signs [6-8]. In addition, it has been shown that women who were self referred with symptoms of chest pain were more likely that men to show CHD symptoms that might also be caused by an anxiety disorder [9].

Since the late 1980s there has been a rising concern that in patients, as far as CHD is concerned, women might be treated and managed differently than men. Healy et al. coined the term 'Yentl Syndrome' reminding their readers that a women with symptoms suspicious of CHD has to behave like a man in order to receive the same diagnostic work up and treatment [10]. In the following years many reports seemed to support the observation that men are often treated earlier, and more aggressively, than women when presenting with similar symptoms [11-13]. However, these findings did not remain uncontested. Results of other authors did not support what was by then termed as "gender bias" in the management of CHD [14-16].

Most of the above cited research has been performed in hospital emergency departments. The few data from a primary care setting were gained from retrospective cross sectional surveys of general physicians' (GPs) medical records and disease registers [17-19].

To our knowledge, ours is the first prospective primary care study investigating gender differences in the management of patients presenting with chest pain. We examined whether there are gender differences in GPs' initial assessment and subsequent management of patients with chest pain, how these differences can be explained, and what underlying mechanisms might be at work.

## Methods

We conducted a cross-sectional diagnostic study with a delayed-type reference standard in a primary care setting [20]. The final diagnosis was established by an expert panel after 6 months of follow up. The main aim of the study was to investigate the diagnostic accuracy of signs and symptoms for chest pain patients with CHD [21]. In this article we report results of a subanalysis with regard to gender differences in GPs' management of chest pain patients.

### Participating GPs and patients

209 GPs in the German state of Hesse were approached by the study team; 74 (35.4%) agreed to participate in the study. Doctors consecutively recruited every attending patient with chest pain, both as presenting complaint and on questioning. Recruitment was staggered in four waves between October 2005 and July 2006; the overall recruitment period lasted 12 weeks for each surgery.

GPs included every patient above 35 years with pain localized in the area between clavicles and lower costal margins, and anterior to the posterior axillary lines. Doctors also recruited during home visits and emergency calls. Patients were eligible irrespective of the acute or chronic nature of their complaints, or of previously known conditions including CHD or related risk factors. Patients whose chest pain had subsided for more than one month, whose chest pain had already been investigated, and/or who came for follow-up for previously diagnosed chest pain, were excluded.

This procedure, like the whole study protocol, was approved by the Ethics Committee of the Faculty of Medicine, University of Marburg. The study complies with the Declaration of Helsinki.

### Data collection

#### Baseline

GPs took a standardized history and performed a physical examination according to a case report form that was piloted and modified accordingly. The report form contained 82 items covering information on basic patient and pain characteristics, accompanying symptoms, and CHD risk factors. GPs recorded their preliminary diagnoses, investigations, and management related to the patients' chest pains. In addition, GPs rated how certain they were regarding their preliminary diagnosis. GPs were also asked to rate the likelihood for CHD in each of their patients. Both measures were recorded on a visual analogue scale ranging from 0-100%.

#### Follow up

Patients were contacted by phone both six weeks and six months after the index consultation. Study assistants, who were blinded to clinical data previously recorded, asked about the course of the patients' chest pain and treatments, including hospitalizations and drugs. Discharge letters from specialists and hospitals were requested by GPs. Where GPs failed to obtain this information, our department requested the necessary documents directly.

#### Precautions against selection bias

Participating GPs were recruited from a network of research practices associated with our department. To GPs we emphasized the importance of recruiting every patient with chest pain irrespective of the presumed likelihood of CHD. GPs were visited at four week intervals to check report forms, recruitment logs and compliance with study procedures. Random audits were performed by searching routine documentation of participating practices to identify cases of chest pain not included in the study.

#### Diagnosis and reference standard

A reference panel consisting of one cardiologist, one GP, and one research associate from the Department of Family Medicine (University of Marburg) reviewed baseline and follow-up data from each patient. They decided on the most likely medical condition having caused the individual patient's chest pain at the time of the index test (delayed type reference standard). In addition, the panel decided whether the collected data supported an indication for urgent hospital admission. The GP's initial diagnosis contributed to the decision made by the panel.

### Statistical analysis

Sample size calculation was based on the primary research question with CHD disease as reference criterion. In low prevalence samples the precision of estimates of sensitivity is critical. To establish a (low) sensitivity of 0.55 with a confidence interval of ± 0.1 we would need 96 patients with CHD. Under the assumption that 8% of patients with chest pain had CHD, 1200 patients had to be recruited. This would allow us to estimate high sensitivities with even more precision, e.g. a CI of the same width for sensitivity = 0.95 would require only 19 patients with CHD [22].

For univariate analyses we calculated proportions and diagnostic odds ratios (OR) for selected items. The Chi-Square test was used for univariate comparisons of categorical data. Fisher's exact test was used when the nominator was equal or below five. The Mann-Whitney U test was used to compare continuous data for significant gender differences.

As this is an explorative study including comparisons between different variables, calculation of the significant p-value took into account the 6 outcomes: p < 0.01 was considered to provide evidence of an association, while p < 0.05 was considered to indicate a possible association [23]. Multivariate logistic regression analysis was performed to adjust the influence of gender on sociodemographic and disease-related differences between women and men. The dependent variables were CHD and the indication for urgent hospital admission. Odds ratios (OR) and 95%-confidence intervals were calculated. In order to adjust for the typicality of chest pain (specific combination of symptoms and signs at patient level), we used the Marburg CHD score, which has been developed and validated as an effective tool for ruling out CHD in patients presenting with chest pain at a primary care level [24]. We used the Mann-Whitney U test to test for significance of gender differences in the distribution of score values. Analyses were performed with SPSS software version 15.0.

## Results

### GPs and patients characteristics

**Figure 1:**
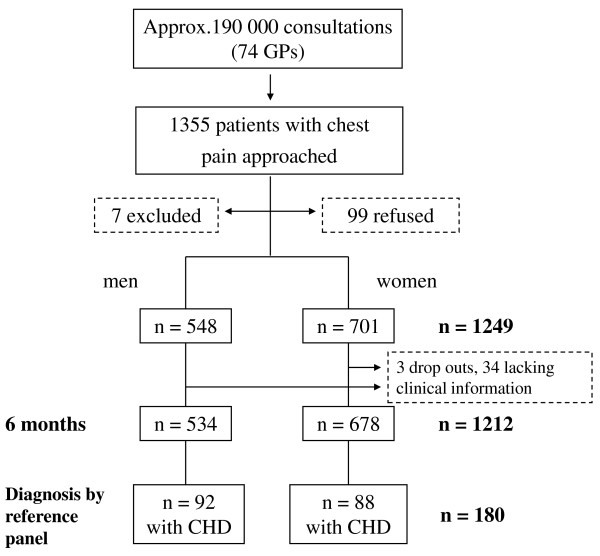
**Patient flow**.

Practices located in urban areas were 63.5%, and 67% of the participating 74 GPs were male with a mean age of 49 years. Figure 1 describes the patient flow. GPs encountered around 190 000 patients during the study period, including 1355 patients with chest pain. Seven patients (0.005%) did not meet the inclusion criteria and 99 (0.07%) refused to participate in the study. GPs returned valid case report forms for 1249 patients (92.2%). Among these were 548 men (43.9%) and 701 women(56.1%) (T0). Although 60 (0.05%) cases were lost to follow-up and 11 (0.009%) died, these 71 (0.06%) provided enough information to be judged by the reference committee. Three early drop outs were not included. For 34 (0.02%) cases follow-up information was incomplete or ambiguous so that no final diagnosis could be made. Therefore, at T1 (6 months) we analyzed 1212 patients (534 men and 678 women) for the etiology of their chest pain; of those 180 (14.9%) patients (92 men and 88 women) were diagnosed as having CHD.

GPs knew the vast majority of both male and female patients from former consultations (93.7% women, 90.3% men, p = 0.03). Most patients quoted chest pain as reason for the actual consultation (88.4% women, 86.6% men, p = 0.29), and nearly half of the patients had acute chest pain at the time of consultation (28.8% women, 30.7% men, p = 0.43).

### Prevalence of CHD in women and men and certainty of diagnosis

In 1212 patients (678 women and 534 men) a diagnostic classification of the underlying reason of chest pain was possible and CHD was confirmed by the reference panel in 88 (13.0%) women and in 92 (17.2%) men. In terms of proportions, there was a significant gender difference (p = 0.04) with men having a higher CHD prevalence in the study sample.

GPs did not show significant gender related differences when rating the diagnostic certainty of their presumed diagnosis. Diagnostic certainty was 85.0% for women (25%; 75% quartile: 70.0%; 90.0%) and 80.0% (25%; 75% quartile: 70.0%; 90.0%) for men (p = 0.21).

### Management of patients with chest pain

Table 1 lists six process indicators (two referring to diagnostic assessment and four to management) initiated by the GP when a patient presented with chest pain. Although GPs tended to rate male patients as having a higher CHD probability than female patients, there are no significant gender differences in the assumption that CHD or Acute Coronary Syndrome (ACS) might be the underlying reason for the patient's chest pain. However, there are gender differences in conducting an exercise test, and in making a decision for hospital admission.

**Table 1:** GPs' assumptions and management of chest pain patients by gender

GP assumption/procedure initiated by GP during index consultation			
	Patients presenting with chest pain (n = 1249)		
	
	Women (n = 701)	Men (n = 548)	p-value
GP assumes CHD being the cause of chest pain - n(%)	126 (18.0)	119 (21.7)	0.10
Exercise test - n (%)	29 (4.1)	40 (7.3)	**0.02**
Referral cardiologist - n (%)	77 (11.0)	67 (12.2)	0.50
Exercise test or referral cardiologist - n (%)	104 (14.8)	104 (19.0)	0.05
GP assumes ACS being the cause of chest pain - n(%)	24 (3.4)	20 (3.6)	0.83
Hospital admission - n (%)	20 (2.9)	36 (6.6)	**< 0.01**

### Appropriateness of GPs' decisions

Table 2 shows the six process indicators against the two reference diagnoses. There are no gender differences for all process indicators in the patient group with CHD, or an indication for urgent hospital admission. In the group where no CHD was diagnosed, more men received an exercise test alone or, in combination with, a referral to the cardiologist. More men were also admitted to a hospital when there was no indication to do so.

**Table 2:** Appropriate decisions in regard to 6 process indicators

	CHD			No CHD		
	
	Women (n = 88)	Men (n = 92)	p-value	Women (n = 608)	Men (n = 450)	p-value
GP assumes CHD - n (%)	59 (67.0)	64 (69.6)	0.72	67 (11.0)	53 (11.8)	0.71
Exercise test - n (%)	3 (3.4)	7 (7.6)	0.22	26 (4.3)	33 (7.3)	**0.03**
Referral cardiologist - n (%)	24 (27.3)	19 (20.7)	0.30	53 (8.7)	47 (10.4)	0.34
Exercise test or referral cardiologist - n (%)	27 (30.7)	26 (28.3)	0.72	79 (13.0)	80 (17.8)	**0.03**

	**Indication for urgent hospital admission**			**No Indication for urgent hospital admission**		
	
	**Women (n = 37)**	**Men (n = 33)**	**p-value**	**Women (n = 505)**	**Men (n = 663)**	**p-value**

GP assumes ACS - n (%)	14 (42.4)	14 (37.8)	0.70	6 (0.9)	10 (2.0)	0.12
Hospital admission - n (%)	12 (36.4)	18 (48.6)	0.30	8 (1.2)	18 (3.6)	**< 0.01**

The horizontal part of the table lists our 6 process indicators reflecting diagnostic assumptions and decisions taken by GPs. The vertical columns show the decision of the reference panel in regard to CHD stratified by gender.

### Diagnostic assumptions and management procedures adjusted for different models

Table 3 compares univariate ORs of the six process indicators with two different logistic regression models adjusting for potential confounders. In the first model (middle column) gender was adjusted for age, different cardiac risk factors, previous diseases, and the final reference diagnosis. For exercise testing and hospital admission, gender differences remained significant.

**Table 3:** Gender differences (OR: female vs. male patients) of 6 process indicators adjusted for different models (n = 1249; OR > 1: more frequent with male patients)

	Gender (univariate)		**Gender (adj. for age, cardiac risk factors)**^**a**^		**Gender (adj. for Marburg CHD score)**^**b**^	
	
	OR (95% CI)	p-value	Adj. OR (95% CI)	p-value	Adj. OR (95% CI)	p-value
GP assumes CHD being the cause of chest pain	1.26 (0.96-1.68)	0.10	1.25(0.89-1.76)	0.21	0.79(0.49-1.29)	0.35
Exercise test	1.83 (1.12-2.98)	**0.02**	1.96 (1.18-3.24)	**< 0.01**	1.21(0.62-2.36)	0.57
Referral cardiologist	1.13(0.80-1.60)	0.50	1.03(0.72-1.48)	0.87	0.73(0.45-1.18)	0.20
Exercise test or referral cardiologist	1.35(1.00-1.81)	0.05	1.31 (0.96-1.78)	0.09	0.88(0.59-1.32)	0.54
GP assumes ACS being the cause of chest pain	1.56 (0.85-2.85)	0.15	1.70 (0.81-3.58)	0.16	1.17(0.43-3.18)	0.76^c^
Hospital admission	2.39(1.37-4.19)	**< 0.01**	3.45(1.76-6.78)	**< 0.01**	2.30(0.99-5.30)	0.05^c^

The following variables were selected as potential confounders for multivariable analysis (binary Logistic regression, inclusion of all variables):

^a ^age (years), CHD (as reference diagnosis for process indicators 1-4), indication for urgent hospital admission (as reference diagnosis for process indicators 5 and 6), known clinical vascular disease, hyperlipidemia, diabetes mellitus, smoking, hypertension, obesity, positive family history for CHD. Except age which was coded continuously (35-99), all other predictors were binary coded (0 or 1).

^b ^results of the Marburg CHD score for each patient, CHD (as reference diagnosis for process indicators 1-4), indication for urgent hospital admission (as reference diagnosis for process indicators 5 and 6). The Marburg CHD Score was coded categorical (0-5 points).

^c ^values should be interpreted with caution as the Marburg CHD score discriminates better for patients with chronic stable CHD

As a next step, we adjusted the second regression model for the typicality of chest pain to see whether these differences would persist by using the Marburg CHD score together with the reference diagnosis CHD. As shown in Table 4, men presenting with chest pain show, on average, significantly higher score values than women, i.e., they present with more clinical symptoms and signs suggestive of CHD. The same applies for the subgroup of patients that were finally diagnosed as having CHD. After adjusting for this specific combination of symptoms and signs in the individual patient, gender differences with regard to the exercise test ceased to exist. There was also a drop in the OR for hospital admission with a marginally significant p-value of 0.05.

**Table 4:** The Marburg CHD score as proxy indicator for the typicality of chest pain: gender distribution for different score values in all patients and patients with CHD

	All patients with chest pain			Patients with ref. diagnosis CHD		
	
	Women (n = 465)	Men (n = 356)	p-value	Women (n = 46)	Men (n = 57)	p-value
**Score 0**	59 (12.7%)	36 (10.1%)		1 (2.2%)	0 (0.0%)	
**Score 1**	129 (27.7%)	78 (21.9%)		1 (2.2%)	0 (0.0%)	
**Score 2**	165 (35.5%)	116 (32.6%)	**<0.01**	16 (34.8%)	17 (29.8%)	**0.01**
**Score 3**	88 (18.9%)	91 (25.6%)		18 (39.1%)	22 (38.6%)	
**Score 4**	24 (5.2%)	35 (9.8%)		10 (21.7%)	18 (31.6%)	
**Score 5**	8 (1.7%)	16 (4.5%)		7 (15.2%)	14 (24.6%)	

The score ranges from 0-5 points. Each of the following five variables contributes 1 point: age (female≥65, male≥55), known clinical vascular disease, pain worse on exertion, patient assumes cardiac origin of pain, pain not reproducible by palpation

### An explanatory model

The key contents of Tables 1, 2, 3 and 4 can be combined to a model for differential management of both genders in cardiac care: In the general population men show on average a higher CHD prevalence and a higher lifetime risk of developing CHD [25]. However, more women than men consult their GP with chest pain (see Table 1). The lower consultation threshold in female patients leads to a dilution effect with resulting different typicality in clinical presentation (see Table 4). Biological differences in women (e.g. pathophysiology of atherosclerosis) [26] might act as an additional factor.

GPs tend to assume that CHD is the cause of chest pain more often in male patients (see table 1). Consequently, GPs refer more men for an exercise test and to the hospital (see Table 1). The fact that these differences cease to exist after adjusting for the typicality of chest pain (see Table 3) shows that equal clinical presentation leads to identical management. Follow up investigations in the group of patients that were not diagnosed with CHD show that there is overinvestigation in men, rather than underinvestigation in women (see Table 2).

## Discussion

We developed a model to explain gender differences in GPs' initial assessment and subsequent management of patients presenting with chest pain in a primary care setting. By defining different process indicators and adjusting these for age, CHD prevalence, underlying risk factors, and the typicality of chest pain in the individual patient, we tried to give an explanation for these differences including the underlying mechanisms at work.

To our knowledge this is the first prospective study with a sufficient sample size to allow investigating gender differences of chest pain in primary care. Strengths of this study are the large GP-based consecutive sample which was highly representative, the prospective design, and small drop out rates. Different study procedures, including random audits, reduced the possibility of selection bias. We did not interfere with the work-up provided by participating GPs, and an interdisciplinary team provided a precise diagnosis as a reference standard.

Part of the analysis was based on small numbers so that existing gender differences might not have reached statistical significance level. Especially the number of patients referred for exercise test and admitted to hospital are small and conclusions need to be drawn with caution. In addition, GPs might have considered an exercise test as a less appropriate examination in women than in men and might have ordered as a consequence this investigation less in women. GPs did also have prior knowledge of more women patients than men. However, it is unlikely that the small difference of 3.4% is clinically relevant in regard to the observed results; it only reached statistical significance because of our large study sample.

There are limitations for using the Marburg CHD score as a proxy indicator for the typicality of the individual patient's chest pain. The score is primarily designed for patients presenting with chronic stable CHD; therefore it is of less value to adjust for urgent hospital admission. In addition, not all score variables reflect clinical conditions that can be related to the typicality of chest pain. The Marburg CHD score has the be regarded as the best option available and results need to be interpreted with caution.

In general, the participating GPs showed a high certainty of their presumed diagnosis, but tended to rate male patients as having a higher CHD probability than female patients. This is supported by findings of a study conducted by Schulman et al. who used a computerized survey instrument to assess physicians' recommendations for managing chest pain and where more male patients were rated as having CHD [27]. However, in our study sample there was a significant gender difference for CHD as reason for the patients' chest pain with men having a higher prevalence than women. This corresponds with the results of representative epidemiological primary care data that show higher CHD prevalence for men [28]. GPs' diagnostic assumptions were therefore in line with the underlying epidemiology of CHD, both observed in our sample and at a national level.

GPs apply different management strategies for chest pain patients ranging from 'wait and see' to urgent hospital admission. We tried to capture these follow up decisions by defining different process indicators. In our sample, GPs sent more men for exercise testing and referred more men to a hospital. After adjusting for potential confounders like age, cardiac risk factors, and the reference diagnosis CHD, these gender differences remained. As a consequence, neither the different CHD prevalence in women and men, nor differences in age or in cardiac risk profile, can serve as an explanation for the GPs' management decisions. Crilly et al. found in a cross-sectional study systematic gender differences in the clinical management of patients with angina in primary care, including a higher rate of exercise tests in men [17]. Two further cross sectional studies conducted in a primary care context showed gender differences in secondary CHD prevention, with men being more likely to be treated with aspirin and statins [18,19]. In contrast to our study sample, the above mentioned studies looked only at patients with an established diagnosis of CHD.

Most studies looking at gender differences in the management of CHD have been conducted in emergency departments mainly including patients with ACS. Among others, findings have been that women are less frequently admitted to the Intensive Care Unit and are less likely to undergo invasive cardiac procedures [11,13,29-32]. However, these findings do not necessarily mean there is a gender bias towards women. They could also reflect overuse of diagnostic and therapeutic procedures in men [11]. Findings of other studies did not support gender differences in the consequent management of patients with CHD or ACS [14-16,33-35]. In a critical analysis, Green and Ruffin remarked that differences in treatment by sex may be a practice variation phenomenon rather than uniform bias [36]. Our data support the above mentioned observations.

It has been argued that CHD presents clinically less typical in women than in men [26,37]. When we used the Marburg CHD score in order to adjust for the typicality of symptom presentation in women vs. men, gender differences for all process indicators ceased to exist [24].

It is an interesting observation that, in our sample, women with CHD did not seem to have any disadvantage from presenting less typical than men. When looking at the subgroup of chest pain patients with CHD as final diagnosis, women and men seemed to receive the same management (see table 2). However, there were contrasting results in chest pain patients where another etiology than CHD was finally diagnosed. For this group our data indicate an overuse of diagnostic procedures like the exercise test and a lower threshold of hospital admission in men. Being confronted with diagnostic uncertainty in the diagnosis of chest pain together with the fear of overseeing serious cardiac disease, a possible explanation could be that GPs have, in general, a low threshold for initiating further diagnostic procedures in all patients where they suspect CHD. While it has so far been argued that women with CHD show out of biological reasons a less typical clinical presentation than men [26], we propose as an additional explanation a gender related difference in the utilization pattern of medical services: women contact earlier and with less typical symptoms their GP than men. This is supported by the findings of different studies which show differences in self perception and symptom reporting between women and men. Women rated their pain as more intense using more affective words and report more often bodily symptoms than men [38-41]. All these aspects might contribute to a lower threshold in women to consult a GP for further investigations. Because of this lower utilization threshold, women present with less typical symptoms and are consequently less referred. As GPs tend to assume a higher CHD likelihood for men [42], and therefore refer more men for further investigations, it might, paradoxically, be just this mechanism that leads to fewer unnecessary investigations in women.

## Conclusions

In summary there are gender differences in GPs' management decisions in patients presenting with chest pain. While these differences can not be explained by differences in age, CHD prevalence, and underlying risk factors, the less typical symptom presentation in women might be an underlying factor. A lower utilization threshold resulting in a dilution effect together with contributing biological mechanisms in the individual patient might explain these differences in typicality. However this does not seem to result in suboptimal management in women but rather in overuse of services for men.

As our conclusions are based partly on very small numbers we consider them rather hypothesis generating. Larger studies will be necessary to prove our proposed model explaining gender differences in GPs' management of chest pain patients.

## Competing interests

Conflict of Interests: JRS acts as scientific advisor for MSD and ESSEX.

All other authors do not declare any competing interests.

## Authors' contributions

NDB formulated the research question, designed the study and supervised its conduct together with ACS. NDB, EB, JH, ACS, MAH, HK, JRS, KK and SB were involved in acquisition, analysis and interpretation of data. SB drafted the article; NDB, EB, JH, ACS, MAH, HK, JRS and KK revised it critically. SB had full access to all of the data in the study and takes responsibility for the integrity of the data and the accuracy of the data analysis. All authors approved the final manuscript.

## Pre-publication history

The pre-publication history for this paper can be accessed here:

http://www.biomedcentral.com/1471-2296/12/45/prepub

## References

[B1] Svavarsdottir AE Jonasson MR Gudmundsson GH Fjeldsted K Chest pain in family practice. Diagnosis and long-term outcome in a community setting CanFamPhysician 1996 42 1122 1128 PMC2146490 8704488

[B2] Nilsson S Scheike M Engblom D Karlsson LG Molstad S Akerlind I Ortoft K Nylander E Chest pain and ischaemic heart disease in primary care BrJ GenPract 2003 53 490 378 382 PMC1314597 12830565

[B3] Verdon F Burnand B Herzig L Junod M Pecoud A Favrat B Chest wall syndrome among primary care patients: a cohort study BMC Fam Pract 2007 12 8 51 10.1186/1471-2296-8-51 PMC2072948 17850647

[B4] Buntinx F Knockaert D Bruyninckx R de Blaey N Aerts M Knottnerus JA Delooz H Chest pain in general practice or in the hospital emergency department: is it the same? FamPract 2001 18 6 586 589 10.1093/fampra/18.6.586 11739341

[B5] Klinkman MS Stevens D Gorenflo DW Episodes of care for chest pain: a preliminary report from MIRNET. Michigan Research Network J FamPract 1994 38 4 345 352 8163958

[B6] Bösner S Haasenritter J Hani MA Keller H Sönnichsen AC Karatolios K Schaefer JR Baum E Donner-Banzhoff N Gender differences in presentation and diagnosis of chest pain in primary care BMC Fam Pract 2009 10 1 79 10.1186/1471-2296-10-79 20003406 PMC2801475

[B7] Arslanian-Engoren C Patel A Fang J Armstrong D Kline-Rogers E Duvernoy CS Eagle KA Symptoms of men and women presenting with acute coronary syndromes AmJCardiol 2006 98 9 1177 1181 10.1016/j.amjcard.2006.05.049 17056322

[B8] Kimble LP McGuire DB Dunbar SB Fazio S De A Weintraub WS Strickland OS Gender differences in pain characteristics of chronic stable angina and perceived physical limitation in patients with coronary artery disease Pain 2003 101 1-2 45 53 10.1016/S0304-3959(02)00319-6 12507699 PMC4310562

[B9] Carmin CN Ownby RL Wiegartz PS Kondos GT Women and non-cardiac chest pain: gender differences in symptom presentation Arch Womens Ment Health 2008 11 4 287 293 10.1007/s00737-008-0021-x 18592345 PMC2574964

[B10] Healy B The Yentl syndrome NEnglJMed 1991 325 4 274 276 10.1056/NEJM199107253250408 2057027

[B11] Ayanian JZ Epstein AM Differences in the use of procedures between women and men hospitalized for coronary heart disease N Engl J Med 1991 325 4 221 225 10.1056/NEJM199107253250401 2057022

[B12] Steingart RM Packer M Hamm P Coglianese ME Gersh B Geltman EM Sollano J Katz S Moye L Basta LL Sex differences in the management of coronary artery disease. Survival and Ventricular Enlargement Investigators NEnglJMed 1991 325 4 226 230 10.1056/NEJM199107253250402 2057023

[B13] Shaw LJ Miller DD Romeis JC Kargl D Younis LT Chaitman BR Gender differences in the noninvasive evaluation and management of patients with suspected coronary artery disease AnnInternMed 1994 120 7 559 566 10.7326/0003-4819-120-7-199404010-00005 8116993

[B14] Bickell NA Pieper KS Lee KL Mark DB Glower DD Pryor DB Califf RM Referral patterns for coronary artery disease treatment: gender bias or good clinical judgment? AnnInternMed 1992 116 10 791 797 10.7326/0003-4819-116-10-791 1567093

[B15] Krumholz HM Douglas PS Lauer MS Pasternak RC Selection of patients for coronary angiography and coronary revascularization early after myocardial infarction: is there evidence for a gender bias? AnnInternMed 1992 116 10 785 790 10.7326/0003-4819-116-10-785 1567092

[B16] Mark DB Shaw LK DeLong ER Califf RM Pryor DB Absence of sex bias in the referral of patients for cardiac catheterization NEnglJMed 1994 330 16 1101 1106 10.1056/NEJM199404213301601 8133852

[B17] Crilly M Bundred P Hu X Leckey L Johnstone F Gender differences in the clinical management of patients with angina pectoris: a cross-sectional survey in primary care BMCHealth ServRes 2007 7 142 10.1186/1472-6963-7-142 PMC2034556 17784961

[B18] Carroll K Majeed A Firth C Gray J Prevalence and management of coronary heart disease in primary care: population-based cross-sectional study using a disease register JPublic Health Med 2003 25 1 29 35 10.1093/pubmed/fdg007 12669915

[B19] Hippisley-Cox J Pringle M Crown N Meal A Wynn A Sex inequalities in ischaemic heart disease in general practice: cross sectional survey BMJ 2001 322 7290 832 10.1136/bmj.322.7290.832 11290638 PMC30561

[B20] Knottnerus JA Muris JW Assessment of the accuracy of diagnostic tests: the cross-sectional study JClinEpidemiol 2003 56 11 1118 1128 10.1016/s0895-4356(03)00206-3 14615003

[B21] Bösner S Becker A Abu Hani M Keller H Sonnichsen AC Haasenritter J Karatolios K Schaefer JR Baum E Donner-Banzhoff N Accuracy of symptoms and signs for coronary heart disease assessed in primary care Br J Gen Pract 2010 60 575 246 257 10.3399/bjgp10X502137 PMC2880766 20529488

[B22] Zhou XH Obuchowski NA McClish DK Statistical Methods in Diagnostic Medicine 2002 New York: John Wiley

[B23] Bland JM Altman DG Multiple significance tests: the Bonferroni method BMJ 1995 310 6973 170 7833759 10.1136/bmj.310.6973.170 PMC2548561

[B24] Bösner S Haasenritter J Becker A Karatolios K Vaucher P Gencer B Herzig L Heinzel-Gutenbrunner M Schaefer JR Abu Hani M Keller H Sönnichsen AC Baum E Donner-Banzhoff N Ruling out coronary artery disease in primary care:development and validation of a simple prediction rule CMAJ 2010 182 12 1295 1300 10.1503/cmaj.100212 20603345 PMC2934794

[B25] Rosamond W Flegal K Furie K Go A Greenlund K Haase N Hailpern SM Ho M Howard V Kissela B Heart disease and stroke statistics--2008 update: a report from the American Heart Association Statistics Committee and Stroke Statistics Subcommittee Circulation 2008 117 4 e25 146 18086926 10.1161/CIRCULATIONAHA.107.187998

[B26] Bairey Merz CN Shaw LJ Reis SE Bittner V Kelsey SF Olson M Johnson BD Pepine CJ Mankad S Sharaf BL Insights from the NHLBI-Sponsored Women's Ischemia Syndrome Evaluation (WISE) Study: Part II: gender differences in presentation, diagnosis, and outcome with regard to gender-based pathophysiology of atherosclerosis and macrovascular and microvascular coronary disease JAmCollCardiol 2006 47 3 Suppl S21 S29 10.1016/j.jacc.2004.12.084 16458167

[B27] Schulman KA Berlin JA Harless W Kerner JF Sistrunk S Gersh BJ Dube R Taleghani CK Burke JE Williams S The effect of race and sex on physicians' recommendations for cardiac catheterization NEnglJMed 1999 340 8 618 626 10.1056/NEJM199902253400806 10029647

[B28] Wittchen HU Glaesmer H Marz W Stalla G Lehnert H Zeiher AM Silber S Koch U Bohler S Pittrow D Cardiovascular risk factors in primary care: methods and baseline prevalence rates--the DETECT program Curr Med Res Opin 2005 21 4 619 613 10.1185/030079905X38187 15899112

[B29] Clarke KW Gray D Keating NA Hampton JR Do women with acute myocardial infarction receive the same treatment as men? BMJ 1994 309 6954 563 566 7916228 10.1136/bmj.309.6954.563 PMC2541441

[B30] Nante N Messina G Cecchini M Bertetto O Moirano F McKee M Sex differences in use of interventional cardiology persist after risk adjustment JEpidemiolCommunity Health 2009 63 3 203 208 10.1136/jech.2008.077537 PMC2635953 19052034

[B31] Chang AM Mumma B Sease KL Robey JL Shofer FS Hollander JE Gender bias in cardiovascular testing persists after adjustment for presenting characteristics and cardiac risk AcadEmergMed 2007 14 7 599 605 10.1197/j.aem.2007.03.1355 17538080

[B32] Roger VL Farkouh ME Weston SA Reeder GS Jacobsen SJ Zinsmeister AR Yawn BP Kopecky SL Gabriel SE Sex differences in evaluation and outcome of unstable angina JAMA 2000 283 5 646 652 10.1001/jama.283.5.646 10665705

[B33] Williams RI Fraser AG West RR Gender differences in management after acute myocardial infarction: not 'sexism' but a reflection of age at presentation JPublic Health (Oxf) 2004 26 3 259 263 10.1093/pubmed/fdh159 15454593

[B34] Setoguchi S Solomon DH Levin R Winkelmayer WC Gender differences in the management and prognosis of myocardial infarction among patients > or = 65 years of age Am J Cardiol 2008 101 11 1531 1536 10.1016/j.amjcard.2008.02.033 18489928

[B35] Boccardi L Verde M Gender differences in the clinical presentation to the emergency department for chest pain ItalHeart J 2003 4 6 371 373 12898800

[B36] Green LA Ruffin MT A closer examination of sex bias in the treatment of ischemic cardiac disease JFamPract 1994 39 4 331 336 7931110

[B37] Swahn E The care of patients with ischaemic heart disease from a gender perspective EurHeart J 1998 19 12 1758 1765 10.1053/euhj.1998.1205 9886717

[B38] Granot M Goldstein-Ferber S Azzam ZS Gender differences in the perception of chest pain JPain SymptomManage 2004 27 2 149 155 10.1016/j.jpainsymman.2003.05.009 15157039

[B39] D'Antono B Dupuis G Fleet R Marchand A Burelle D Sex differences in chest pain and prediction of exercise-induced ischemia CanJCardiol 2003 19 5 515 522 12717487

[B40] Barsky AJ Peekna HM Borus JF Somatic symptom reporting in women and men JGenInternMed 2001 16 4 266 275 10.1046/j.1525-1497.2001.00229.x PMC1495200 11318929

[B41] Wool CA Barsky AJ Do women somatize more than men? Gender differences in somatization Psychosomatics 1994 35 5 445 452 7972659 10.1016/S0033-3182(94)71738-2

[B42] Arber S McKinlay J Adams A Marceau L Link C O'Donnell A Patient characteristics and inequalities in doctors' diagnostic and management strategies relating to CHD: a video-simulation experiment SocSciMed 2006 62 1 103 115 10.1016/j.socscimed.2005.05.028 16002197

